# PuzzleWalk: A theory-driven iterative design inquiry of a mobile game for promoting physical activity in adults with autism spectrum disorder

**DOI:** 10.1371/journal.pone.0237966

**Published:** 2020-09-10

**Authors:** Bogoan Kim, Daehyoung Lee, Aehong Min, Seungwon Paik, Georgia Frey, Scott Bellini, Kyungsik Han, Patrick C. Shih

**Affiliations:** 1 Department of Software and Computer Engineering, Ajou University, Suwon, Republic of Korea; 2 Department of Artificial Intelligence, Ajou University, Suwon, Republic of Korea; 3 Department of Applied Human Sciences, University of Minnesota Duluth, Duluth, Minnesota, United States of America; 4 Department of Informatics, Indiana University, Bloomington, Indiana, United States of America; 5 Department of Kinesiology, Indiana University, Bloomington, Indiana, United States of America; 6 Department of Counseling and Educational Psychology, Indiana University, Bloomington, Indiana, United States of America; University of Massachusetts Amherst, UNITED STATES

## Abstract

Primary symptoms of adults with autism spectrum disorder (ASD), such as pervasive social deficits in social interaction and communication, cause adults with ASD to adopt a sedentary lifestyle. Meanwhile, gamified and behavioral theory-based interventions have been shown to improve physical activity in a fun and unobtrusive way. In this paper, we describe the iterative design inquiry process of *PuzzleWalk*, a gamified, physical activity-promoting mobile app designed for adults with ASD. We report the design rationales and lessons learned across four user-centered design phases with ASD experts and adults with ASD, including user requirement gathering, iterative participatory design, usability evaluation, and field deployment. The design insights generated from this work could inform future research focusing on designing sociotechnical systems, games, and interventions for people with ASD.

## 1 Introduction

Autism Spectrum Disorder (ASD) is a pervasive neurodevelopmental disorder characterized by impairments in social interaction along with unusual patterns of restricted, repetitive behaviors [1]. According to a report from the Centers for Disease Control and Prevention (CDC) in 2018, the estimated prevalence of ASD is about 1-in-59 children in the US and has steadily increased over the past two decades. The trend is likely to continue [2]. For this reason, research on how to best support children with ASD has blossomed in the past couple of decades [3, 4].

However, adults with ASD have not received similar attention when compared to children with ASD, and relatively little research on the prognosis, outcomes, or effective interventions for adults with ASD [5, 6] has been conducted. Many medical experts in ASD (e.g., developmental pediatricians) stop seeing autistic patients when they reach the age of majority, and only few physicians receive training for caring adults with ASD [7]. The lack of attention in research, treatment, and care could be one reason for the significantly poorer health and well-being of the adults with ASD [8, 9]. The majority of adults with ASD experience comorbid psychological disorders, such as anxiety [1, 10], and those disorders primarily occur or worsen during the transition to adulthood, since many adults with ASD tend to lack the skills and resources to successfully achieve their desires, such as participation in employment and social relationships [11]. Hypoactive diseases, such as obesity, hyperlipidemia, and hypertension, which are caused by the lack of physical activity (PA), are especially prevalent [12–14], and PA in ASD adults decreases as the age increases [15].

Research has demonstrated the lack of PA in ASD adults is highly correlated with increased risk of many psychiatric and medical conditions [16, 17]. However, there are only a few published studies that investigated PA in adults with ASD [18–20], and most existing PA data are based mainly on children and youth with ASD [21]. Although health-related PA guidelines generally recommend that adults should participate in at least 150 minutes of moderate to vigorous PA to get health benefits [22], a proxy report-based study found that adults with ASD exhibit an extremely inactive lifestyle (i.e., being sedentary for an average of 13 hours per day) [18]. Since PA and exercise have been used with some success to treat maladaptive behaviors or poor social skills in people with ASD [19, 20], it is likely that PA could be served as an effective adjunct anxiety treatment as it does for the general population [23].

Mobile apps have been regarded as a promising tool for monitoring and increasing PA when compared to traditional PA or exercise interventions in the general population [24–29]. However, the long-term success of mobile apps in promoting PA behavior change is still questionable and has yet to be properly evaluated [30–32]. In addition, most commercially-available PA apps are not designed based on health behavior theory, such as behavior change techniques (BCTs) [33]. Behavior Change Techniques are systematic procedures consisting of strategic action plans using psychological determinants, such as self-esteem and motivation, designed to change a human’s health behavior [34]. The effectiveness of BCT has been well verified through prior studies in the public health domain [35–39]. Given the essential role of a theory-based approach in verifying scientific, replicable evidence, the effectiveness of numerous available fitness/PA apps in the commercial markets remains unclear.

To develop fitness/PA apps for the ASD population, more considerations on their characteristics are needed, such as their preference for visual reasoning processes [40] and restricted and repetitive behaviors [41, 42]. Furthermore, there have been only few attempts to investigate the effectiveness of mobile technology-guided interventions on leveraging PA in individuals with ASD. A pilot study with a small sample showed a potential usability of a visualized mobile system in teaching various fundamental gross motor skills to children with ASD as an effective alternative to traditional exercise interventions [43]. However, this system is limited to children with ASD and needs the substantial assistance of a caregiver or instructor for proper execution. As a scaffolding mechanism, it is crucial for an app to focus on encouraging PA in adults with ASD without any initial assistance and social requirements. Likewise, mobile health interventions for increasing PA in real-world settings, especially for adults with ASD who are required for independent healthy lifestyle, are significantly lacking. According to the recent autism research priorities [44], there is a critical need for more effective treatments over autism-associated health conditions, particularly by adults in real-world settings [8, 45]. Therefore, in this paper, we introduce a mobile app, *PuzzleWalk*, developed through an iterative design process, especially combined with core elements of user-centered design (UCD; survey, interview, and prototyping) with ASD stakeholders based on BCTs (e.g., self-monitoring, visual rewards, feedback on performance, user instruction, and goal-setting) and gamification. The main goal of *PuzzleWalk* is to promote PA with mobile technology-leveraged behavior change interventions. We employed *walking* as a means to operationalize PA in adults with ASD due to their below-average motor skills [46, 47] and a higher risk of being obese than general population [13, 48, 49]. *PuzzleWalk* features a mixture of predictable and sustainable interfaces that are aimed at meeting the technical affinity and expectations of autistic adults with cognitive function based on the findings of a series of design inquiries presented in this paper.

The contributions of this paper are as follows.

- We report design rationales, lessons learned, and salient factors that informed us through a series of design inquiries of a mobile app for adults with ASD.
- We demonstrate the effectiveness of a BCTs-based smartphone application, *PuzzleWalk*, designed through UCD.
- We assess the perception of use and feasibility of *PuzzleWalk* in increasing PA in adults with ASD through a one-month field study.

## 2 Related work

### 2.1 PA intervention through behavior change techniques

A Behavior Change Technique (BCT) is a theory-based method for altering one or more psychological behavior determinants, such as a person’s attitude or self-efficacy [33, 34]. Effective BCTs could include one or a combination of techniques to address health behavior change, and mobile technologies are accessible, time-, and cost-effective for delivering diverse BCTs in real-world settings [36, 50]. Bird et al. [37] conducted a meta-analysis of walking and cycling interventions, and reported that the effectiveness of intervention was associated with integration of self-monitoring with other self-regulatory BCTs. There are currently more than 325,000 apps designed for promoting health and wellness available at major app stores [51]. Although these apps have potential to enhance PA engagement, the majority of them are not designed based on BCTs, and only a few have been evaluated through scientific methods [52, 53].

Conroy et al., [30] analyzed 167 top-ranked apps designed for increasing and/or tracking PA (Android, 38 free and 37 paid; iOS, 43 free and 49 paid) and found that people tended to use multiple apps at once. BCTs also were not widely utilized in top-ranked apps. Without systematically assessing the effectiveness of the PA apps, it is unclear whether any positive health outcomes are derived from the app use, driven by having a strong intrinsic motivation, or other factors [35]. In this work, we incorporate BCTs from existing PA, ASD, and HCI literature in the design of a mobile PA intervention app that is iteratively refined and evaluated by the target users with ASD.

### 2.2 Gamification techniques as health intervention

Gamification is defined as “the use of game design elements in non-game contexts” [54]. Gamified mobile apps utilize game-like elements, such as animated characters, problem-solving, and engaging storylines, to elevate intrinsic motivation. Research has found that gamification can positively influence psychological, physical, and health-related behaviors and outcomes [55, 56]. Gamified mobile apps have been demonstrated to have potential in improving user engagement and retention of target behaviors by providing users with feelings of excitement, fun, and desire [57, 58].

As a PA intervention, Kuramoto et al. [59] developed “Stand Up, Heroes!”, a gamified mobile app for motivating commuters to keep standing on public transportation in Japan. In this system, participants can accumulate experience points to collect equipment items for their avatars by standing during their commute. However, although participants praised the gamification features of the system early in the user study, usage dropped once they collected all of the items. Martin-Niedecken et al. [60, 61] developed “Plunder Planet,” adaptive exergame fitness training for children and adolescents. This game was designed to be played with motion-based controllers through tangible or gesture-based input movements. Through a series of gestures and movements, users can enjoy games by sailing on ships or dodging obstacles while promoting PA at the same time.

Gamification indeed is pervasive in popular commercial health/fitness mobile apps [62]; however, our literature review indicates that there is little scientific evidence to demonstrate their effectiveness and efficacy in improving the level of PA. By following the design inquiry process in our work, we hope to understand the motivations and design rationales of gamified elements that could lead to sustainable PA intervention for adults with ASD.

### 2.3 Mobile technology for autism

There are mobile technologies that help people with ASD and their family learn how to better cope with ASD (e.g., behavioral intervention), overcome challenges (e.g., health management support), and improve their well-being and daily functions (e.g., communication, social, and problem-solving skills) [63, 64]. There have been numerous attempts to leverage mobile technologies to assist them in the areas of education [65, 66] and health [67] in the past decade [63]. For example, Hirano et al. [68] presented “vSked,” a collaborative visual scheduling and reinforcement system designed for classrooms with children with ASD. The system was evaluated with a high level of satisfaction by nine children with ASD. Teachers also praised the system in terms of reducing their effort required for using visual supports by automatically generating records and reports. Escobedo et al. [69] presented “MOSOCO,” the Mobile Social Compass System designed to support social skills of children with ASD. MOSOCO employed the augmented reality (AR) and visual supports of the Social Compass social skills curriculum [70]. This system enabled users to learn social skills in a real-setting through interactive features encouraging eye contact and other social skills (e.g., space and proximity, sharing interests). Due to the high accessibility through mobile devices, social media has also been used as a means of social support. Hong et al. [71] created “SocialMirror” to connect young adults with ASD to their social network to obtain information and advice about their everyday life. This study demonstrated the feasibility of using social media to assist in the successful transition for young adults with ASD.

In addition to smartphone systems, wearable devices have been used to provide better assessment and socialization of people with ASD. Amiri et al. [72] has designed WearSeanse to detects one’s autistic behaviors and to facilitate better clinical assessment. ProCom has been developed to support the social interaction of people with autism by improving their perception of real-time physical proximity in social settings [73, 74]. WELI (Wearable Life) is a wearable app designed to support students with intellectual and developmental disabilities [75]. It provides interventions with personalized notifications to help their preparation, engagement, and collaboration in class. It has been shown that mobile and wearable technologies could have positive impact on adults and children with ASD.

## 3 Overview of research procedure

**Fig 1:**
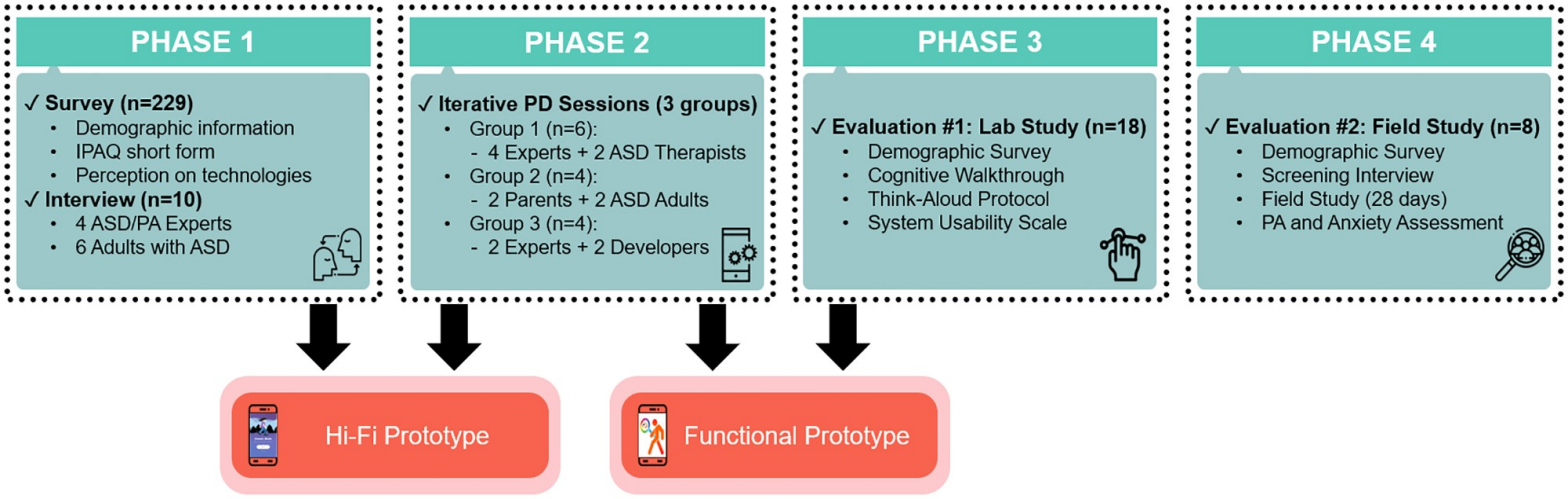
Overall research procedure across four phases of the study. In Phase 2, “PD” = participatory design, “Parents” = parents of a child with ASD, and “Experts” = ASD/PA experts. We held three iterative PD sessions with each group, totaling nine PD sessions.

Our research consists of four iterative design inquiry phases: (1) understanding user requirement and design ideation, (2) iterative participatory design sessions, (3) usability evaluation, and (4) assessment of the system effectiveness and feasibility. Fig 1 shows the overall research procedure.

In Phase 1, we aimed to specify the user target behavior and identified design elements for developing a system to improve the target behavior.

In Phase 2, we gathered feedback through iterative participatory design sessions to finalize a gamified mobile app to increase PA of adults with ASD.

In Phase 3, we recruited 18 participants and assigned them to two groups (i.e., 9 for ASD group, 9 for non-ASD group), and investigated the system validation and usability before releasing the final version of *PuzzleWalk*.

In Phase 4, we conducted a field study of adults with ASD for a month to examine the feasibility of *PuzzleWalk*, a gamified and BCT based mobile app, compared to Google Fit. We employed a mixed method study design in randomized controlled trial and reported the results of eight adults with ASD.

Below, we report each phase of the study in detail and describe the design rationales and lessons learned through the iterative design inquiries in each phase. We denote participant quotes using “PX-YY” to refer to “Phase X-Participant Number YY” (e.g., “P1-15” means “Phase 1-Participant 15”) throughout the remainder of the paper. For each phase of the study, the participants signed an informed consent form and this study was approved by the Indiana University Institutional Review Board.

## 4 Phase 1: Understanding user requirement and design ideation

In the first phase, we aimed to specify user target behaviors and identified design elements for developing a system to improve such behaviors. In particular, we examined how mobile technology affects sedentary behavior and PA of adults with ASD, and what design elements are required to develop a mobile system that sufficiently reflects those characteristics (e.g., predictable and persistent User Interface (UI) for visual learners). To do this, we employed the User-Centered Design (UCD) method. UCD is an iterative design process in which system designers focus on potential users and their requirements in each step of the design process [76, 77]. UCD not only leads to practical guidelines and a set of evaluation criteria of a newly designed system [78] but also calls for stakeholders throughout the design process via a variety of research and design techniques to create highly usable and accessible products [79]. A system designed with UCD methods leverages the user experience and achieves the goal of the system. Involving adults with ASD will benefit us by reflecting user feedback and needs directly into a prototype and accelerating consensus decision making.

In addition, through the literature review of the existing health behavior theories, we identified that PA intervention effectiveness can be enhanced by the integration of self-regulatory BCTs [37]. Thus, we mapped the following BCT principles to specific app functionalities to amplify the effects of *PuzzleWalk* on increasing daily PA engagement for the target population: (1) presenting prompt self-monitoring of performance (i.e., visualizing step-tracking graphs along with the daily, weekly, and monthly step trends), (2) providing instruction on how to perform the target behavior (i.e., providing user guide pages for users to comprehend the key functions of *PuzzleWalk*), (3) providing tangible rewards based on progress toward the successful outcomes (i.e., giving a monetary reward to top three rankers every month using a leaderboard), (4) providing user feedback on performance (i.e., providing applicable tips regarding how to increase PA and reduce sedentary time in a daily living), and (5) prompting users to set up a goal of their daily walking step. Two of the aforementioned BCTs (i.e., visualized health behavior monitoring and goal-setting) were developed together with gamification features. We incorporated validated BCT principles in the initial design of *PuzzleWalk* as our starting point, and iteratively refined *PuzzleWalk* through a series of UCD inquiries.

### 4.1 Survey & interview

We conducted an online self-report survey including 229 adults with ASD to identify their perception of PA and regularly technology use. We recruited survey respondents through several online autism support groups in Wrong Planet (https://wrongplanet.net), Facebook, and Reddit that advocate for autism awareness and other national and international autism organizations such as Autism Speaks (https://www.autismspeaks.org) in English-speaking countries (e.g., U.S.A., Canada, United Kingdom). The inclusion criteria included (1) age between 18-55, (2) presence of an ASD diagnosed by a qualified medical professional, (3) ability to walk without any assistance, and (4) capability to understand the purpose of a study and complete the survey without any assistance.

**Fig 2:**
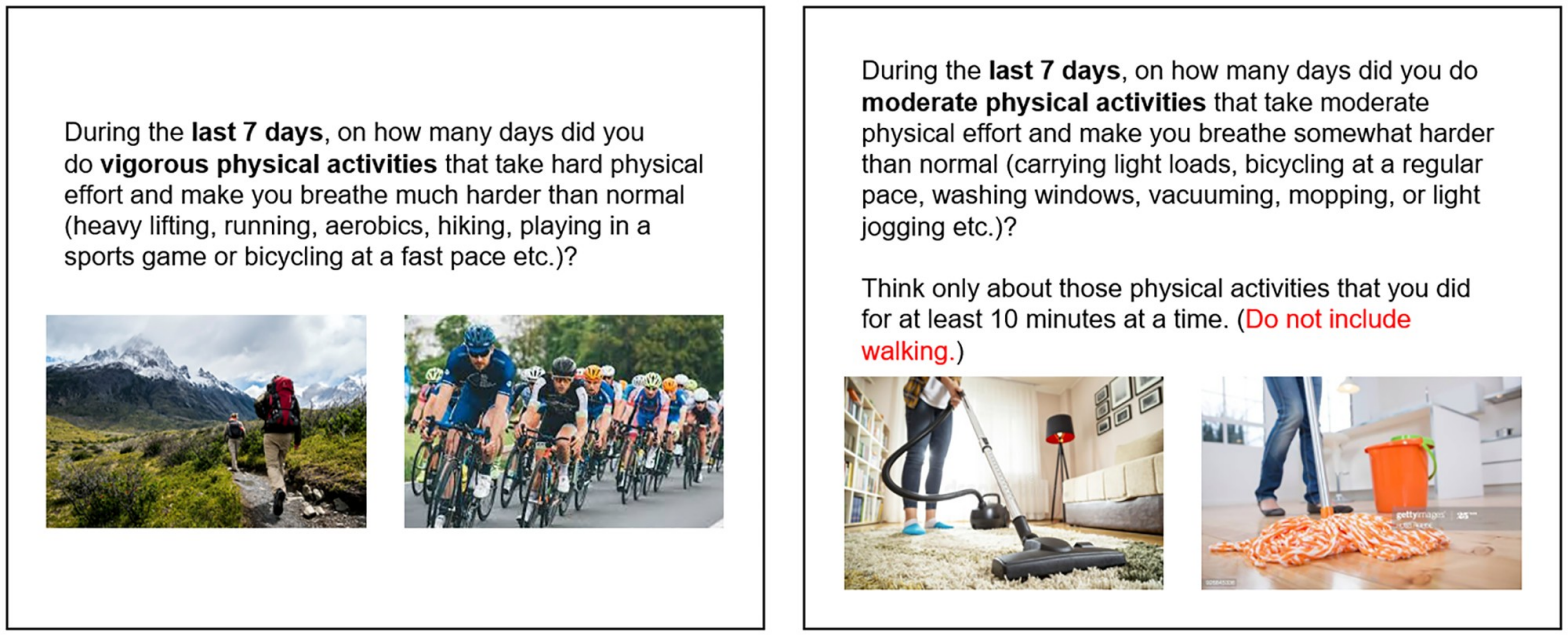
Examples of the modified IPAQ questionnaires used in our research.

The survey was assessed by two survey research experts regarding content validity on the survey’s methodology and readiness. We then conducted a pilot test with six adults with ASD who are not included in our final 229 survey respondents. As a result, we enhanced the readability and comprehension by adding visualized examples to the questionnaires. The finalized survey questionnaire consisted of two primary sections. The first section consisted of the items which examined a respondent’s demographics (e.g., age, sex, education, current autism therapies or interventions, medication use etc.) and the severity of autism symptoms using Autism Spectrum Quotient-10 (AQ 10) [80]. The second section consisted of a total of 34 items (33 selection questions and one open-ended question) that examined the perception of PA (seven items of the International Physical Activity Questionnaire (IPAQ) short form [81]) and the characteristics of technology use (e.g., How much time do you spend on your smartphone each day?; For what purpose(s) do you usually use your technology devices?; How much time did you usually spend using technologies to carry out online games?; How often do you use technology to communicate with your friends?) in adults with ASD. As we identified from the pilot test, we modified the inquiries of the IPAQ by providing more explicit visual examples to increase participants’ understanding (e.g., example activities of each PA intensity; See Fig 2). The open-ended question was asked to collect information about PA barriers in adults with ASD. Of the 802 responses initially collected, we eliminated incomplete or unreliable responses (i.e., survey completion time less than a minimally expected length of time; 15 minutes), leaving 229 viable responses (147 males, 78 females, four other, and age range 18-24; 25%, 25-34; 50%, 35-44; 20%, 45-55; 5%) for the analysis.

We also conducted semi-structured individual interviews with four experts (two ASD experts, two PA experts) and six adults with ASD (three males, avg. age: 26) to develop and implement a pilot survey test and better understand any social and health issues. The average duration of the interview was 35 minutes with note-taking. The two ASD experts have more than 10 years of experience in ASD research in many health settings. During the interviews, we discussed topics mainly pertaining to daily activities, social preferences/challenges, and technology accessibility in adults with ASD (e.g., challenges that adults with ASD face in their daily life).

We evaluated each step of Phase 1 through meetings with each researcher, including the two ASD experts, two PA experts, and six adults with ASD. To analyze the open-ended survey responses and the interview transcripts, three authors of this paper independently coded the data, compared coded results, and resolved any conflicts on the themes among the coders through iterative discussions [82]. We categorized emerged design elements and user requirements obtained from the interview with ASD and PA experts by using an affinity diagram [83]. We arrived at three primary themes—Game design, Rewards, Social.

### 4.2 Phase 1 findings: Lessons learned from survey and interview

From the survey, we confirmed that walking is the major form of their PA (74%, 169 of 299) due to its low barrier in terms of financial cost and accessibility. Furthermore, we confirmed that smartphone usage and quality of life are correlated with sedentary time. Below, we highlighted two findings: (1) inactive lifestyle and (2) tech-savvy.

#### 4.2.1 Inactive lifestyle

The results of the survey indicate that more than 70 percent of the respondents walked fewer than two hours on both weekdays and weekends. Moreover, there was a significantly negative correlation between sedentary time and quality of life (*r* = −0.32, *p* < 0.001) [84]. Based on some of the interview responses, we reaffirmed that adults with ASD have a very high level of sedentary time.

“There is only one study that has reported on PA in adults with ASD, based on proxy reports, shows that this population segment is extremely inactive.”

(P1-1; an PA expert)

“I have difficulty to read people, and I think we are less social and go out less, so we have more time to spend at home.”

(P1-8; an adult with ASD)

We found that adults with ASD prefer to stay at home since getting involved in social activities made them feel anxious, inferior, or fear communication. From the survey, the respondents specified their primary barrier to participating in PA, including physical problems (51%, 116 out of 229), lack of willingness and social skills (27%, 62 out of 229), and anxiety (16%, 37 out of 229). This often results in increased sedentary time and an inactive lifestyle. Thus, adults with ASD need a trigger to boost their motivation and remove intrinsic barriers to increase PA participation. To do this, it would be desirable to design an unobtrusive system that naturally induces and increases PA, considering their anxiety and difficulty in social interaction.

#### 4.2.2 Tech-savvy

From our survey of 229 adults with ASD, we found that the respondents’ average technology usage time is more than six hours per day on both weekdays and weekend. The survey also reported that adults with ASD used their smartphones more than four hours per day. The technology was primarily used for playing games (mean: 145.8 minutes, SD: 126.2 minutes) and other entertainment, such as watching videos, listening to music, and reading e-books (mean: 106.8 minutes, SD: 93.4 minutes). Sedentary time was significantly correlated with technology usage time on both weekdays and weekend (*r* = 0.34, *p* < 0.001) [84]. These results indicate that a majority of respondents feel the importance of technology in their lives and may have prior experience in using (or at least are willing to use) mobile apps such as games.

“People on the spectrum tend to have particular interests which can be pursued on the internet. Even as far as carriers go, people on the spectrum often become coders and so on.”

(P1-3; an ASD expert)

“I think technology is something that is orderly and easy to figure out for people with ASD. It’s something that will always act the same way, and it’s something you can control. It’s something more understandable and orderly.”

(P1-5; an adult with ASD)

As mentioned above, our potential users have a high affinity for technology use. *PuzzleWalk* intends to transform their conventional technology time into a health-related PA time by designing a mobile game that is attractive to adults with ASD and nudging them to engage in an achievable form of PA.

### 4.3 High-fidelity prototype of PuzzleWalk

**Fig 3:**
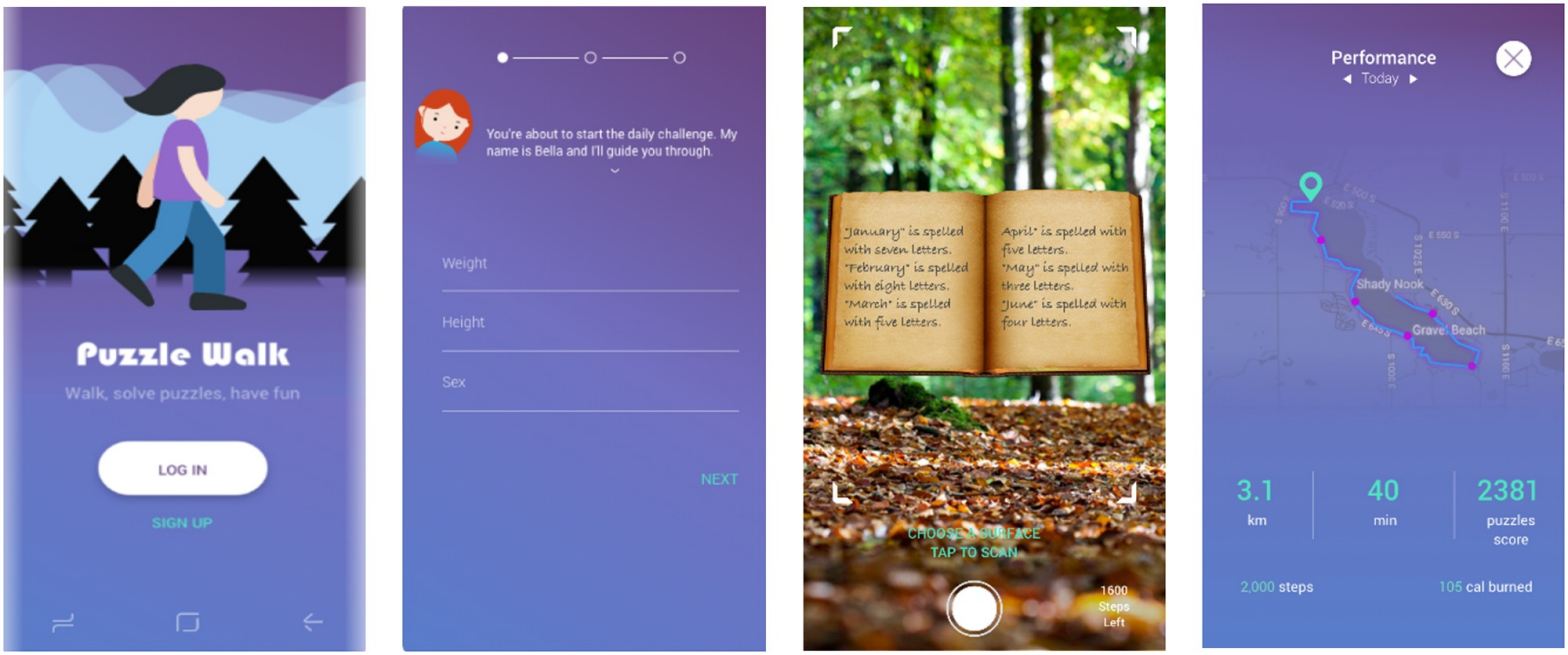
Screenshots of the high-fidelity prototype (e.g., sign up, onboarding, gathering a hint, feedback on performance).

Based on the findings from Phase 1, we derived more concrete user scenarios, and designed the major interaction flow for the high-fidelity prototype (Fig 3). To develop the initial prototype of a mobile app, *PuzzleWalk*, we incorporated the modified version of Integrate, Design, Assess, and Share (IDEAS) framework [85–87]. This framework was devised for developing digital interventions to effectively improve health behaviors. We added several gamification [54] features (i.e., animated characters, problem-solving, and engaging storylines) with the hope of elevating intrinsic motivation and enforce engagement by experiencing excitement and desire [57, 58] to support retention and continuous use. We incorporated design elements extracted through UCD methods in Phase 1. Finally, we developed the first app-based version of *PuzzleWalk* designed to provide effective interventions to increase PA in adults with ASD.

#### 4.3.1 How to play

With the app, a user goes out and navigates a route displayed by *PuzzleWalk*. When a certain number of steps are fulfilled, the user will receive a prompt with an AR hint. With the hint, the user can select to play one of three puzzle types (e.g., word games, numbers quizzes, and shape fold games) based on their preference. After a puzzle is solved, the user needs to navigate another route to play another game.

## 5 Phase 2: Iterative participatory design

From Phase 1, we learned that potential users of *PuzzleWalk* have a significant amount of sedentary time and a high dependence on mobile technology. We also reaffirmed the need for a mobile app that effectively triggers voluntary PAs without asking users (adults with ASD) to be involved in social activities. To improve the high-fidelity prototype, we conducted three parallel iterative participatory design sessions in our laboratory with (1) four ASD/PA experts (10-plus experienced) and two ASD therapists (20-plus experienced), (2) two parents of a child of ASD and two adults with ASD, (3) two ASD/PA experts (10-plus experienced) and two software developers. Through iterative participatory design sessions, we specifically investigated whether mechanisms in solving puzzles were optimized to motivate adults with ASD to “sit less” and “walk more”. These three parallel sessions were conducted iteratively across three different time points, which resulted in a total of nine participatory design sessions being held. The average duration of a session was 45 minutes. All sessions were audio recorded, transcribed, and generated sketches and papers that were photographed.

During each session, the design session chair (experimenter) asked each participant for their views to make all of participants voice their ideas and concerns freely. In the first round of sessions, we showed the participants the high-fidelity prototype and asked them to get familiar with *PuzzleWalk*’s design and main functionalities. In the second round, participants were asked to identify common challenges of *PuzzleWalk* by evaluating and refining revised scenarios, prompts, and the prototype. The participants identified the issues of app navigation, UI readiness, and graphic design including even font size and colors that are deemed attractive for adults with ASD. Finally, participants were asked to evaluate a functional prototype of *PuzzleWalk*.

Three authors of this paper coded the transcripts of the participatory design sessions in Phases 1 and 2 through iterative discussions and consensus building. Gamification, safety, and external motivation emerged to be key components for the sustainable use of *PuzzleWalk*. Lessons learned and detailed design-specific findings informed us what we needed to add, exclude, and supplement to finalize *PuzzleWalk* for adults with ASD. These functionalities were elaborated and refined iteratively with ASD and PA experts involved in both Phases 1 and 2.

### 5.1 Phase 2 findings: Lessons learned from high-fidelity prototype for developing functional prototype

Our participants in Phase 2 felt generally positive about the concept of *PuzzleWalk*, and visually vibrant prototype interfaces because people with ASD are visual learners [40]. Table 1 summarizes revised functionalities mapped with the following highlights—(1) *user sensitivity*, (2) *user safety*, (3) *user engagement*, and (4) *game playability*—to develop the functional prototype of *PuzzleWalk*.

**Table 1:** From high-fidelity to functional prototype: Revised functions based on iterative participatory design.

Finding	Change	Description	Reason
**User Sensitivity**	AR Hint	- Remove AR hint cues to solve puzzles due to sensory issues of target population	- AR hint only promotes PA at the recommended areas where the hint exists and the indoor activity can be overlooked
**User Safety**	Gaming Steps	- Change the procedures of *PuzzleWalk*	- Users might encounter dangerous situations when searching for hints while walking. Thus, we changed the way to play *PuzzleWalk* so that users walk first and then play without walking behavior
	Deactivation Time	- Adding game deactivation time (10 PM–8 AM)	- To avoid possible concerns relating to the nightly smartphone use and its consequent negative effects on sleep problems
**User Engagement**	Walking Step Graph	- Providing step graphs	- Since they like to communicate with visual cues, we changed for user feedback on performance to visualized bar graphs
	Leaderboard	- Providing leaderboard	- To motivate everyday walking on a regular basis
	Monetary Reward	- Giving monetary rewards to top 3 score leaders	
**Game Playability**	Game Types	- Changing to ‘spot the difference’ game	- People with ASD are visual learners, and tend to be more attracted by visualized, animated puzzle games
	Difficulty Levels	- Adding three difficulty levels (i.e., level 1 ∼3)	- To avoid monotony of the game, we rewarded more points when solving more difficult puzzles
	User Guide	- Providing user guide pages about key functions of *PuzzleWalk*	- To enable users to comprehend the key functions of the animated icons and the principle concepts of the gamification techniques between walking steps and gaming time

#### 5.1.1 User sensitivity

Prior research has demonstrated the potentials of utilizing AR for developing environments for diagnostic, therapeutic, and supportive purposes [88, 89]. We initially employed the AR technique in the high-fidelity prototype of *PuzzleWalk* to derive visual attention and sustain user interest. However, there is disagreement among our experts about providing AR hints due to their sensory issues and the characteristics surrounding them. Specifically, some of adults with ASD are sometimes extremely sensitive to environments, such as sight, hearing, and touch [1, 90]. Since minimizing context barriers such as surrounding auditory or visual distractions was pivotal for our design, we concluded that until future research demonstrates the feasibility of AR for people with ASD, the AR technique is not a needed feature to promote their PA, could potentially be distracting, and hinder their use and adoption.

Since users might encounter sensory issues from pop-up AR cues, we excluded the step of obtaining a hint with the AR technique from *PuzzleWalk*. The improved system now involves only two simple steps (i.e., walk and solve puzzles) instead of three (i.e., walk, obtain a hint in AR, and solve puzzles). This change also made the game easier to learn and more intuitive for the users.

#### 5.1.2 User safety

We were aware of the danger of walking while looking at the smartphone through AR-based games as exemplified by Pokémon Go [91, 92]. In our high-fidelity prototype, *PuzzleWalk* included the function of sending a push notification that provided warnings when a user attempts to play the game while walking. The warning message stresses the risk of walking into dangerous objects while using smartphone. However, we received some feedback about lurking dangers such as fatal traffic injuries. To minimize possible concerns, the experts recommended separating the process of PA from solving the puzzles in *PuzzleWalk* by reducing the screen time for adults with ASD when engaging in PA.

We revised two functions to reduce the risk of walking. First, we separated pursuing PA and puzzle solving in *PuzzleWalk*. In the high-fidelity prototype, walking, searching for hints while walking, and solving puzzles were interconnected. Autism experts pointed out that possible concerns may occur in this interconnected process. Thus, the new version prevented dangerous situations by eliminating the process of searching for hints and allowing users to play games without walking behavior.

Additionally, gameplay is deactivated every night between 10pm-8am. Prior research has demonstrated that nightly smartphone use can cause sleep problems in the general population [93]. ASD experts pointed out that the sleep disturbance, which autistic people usually suffer [94], could worsen. To minimize nighttime smartphone use and the subsequent potential negative effects on sleep quality (e.g., delayed sleep onset, reduced sleep duration), we decided to deactivate the puzzle games during typical bedtime to prevent obsessive play.

#### 5.1.3 User engagement

Although the majority of our participants had a positive impression of the interface design, some of them were skeptical about the user engagement in the system. We found that users needed more enticing design elements to encourage them to spend their time on *PuzzleWalk*. Some ASD participants suggested adding visualizations to compare their results as a social component. Also, they were only interested in seeing information relevant to their gaming performance (i.e., gaming time and total score). They felt information of walking distance is distracting since it is unrelated to the overall objective, which is to obtain the highest scores.

Therefore, in our revision of *PuzzleWalk*, we focused on improving user engagement and adding visualizations for performance comparison. We added the following visualization elements to encourage and promote consistent PA engagement. The revised *PuzzleWalk* presents a user’s performance into three types of visualizations, showing a user’s daily, weekly, and monthly walking trend. The revised interface also provides a leaderboard and offers monetary rewards—a $10 e-gift card to top three leaders every month.

#### 5.1.4 Game playability

Our high-fidelity prototype was initially designed to guide a user to a set of recommended locations, where they could find a location-based AR cue upon arriving the destinations. The gaming time is rewarded only when the destination is reached. Based on the feedback from Phase 2, we identified barriers that disturb users’ sustainable engagement. Specifically, *PuzzleWalk* can only be used in good weather conditions at a few preset locations, which not only interrupts the user’s PA promotion but also reduces the user’s engagement to the system.

The types of games included in the high-fidelity prototype were word games, numbers quizzes, and shape fold games combined with AR. However, we changed the game type to “spot the difference” (find different “spots” by comparing two provided pictures) in the functional prototype since the adults with ASD are visual learners [40] and visual supports are highly effective for them [95]. Through this design, we were able to decouple the game with preset locations, and users could also carry out PA indoors or at a location of their choice.

We reached a consensus that the difficulty of a challenge while solving puzzles enables users to have a tension that can increase the user’s enjoyment [96, 97]. Thus, we introduced three difficulty levels (i.e., easy: 30 points; moderate: 40 points; difficult: 50 points) to enhance sustainability.

In addition, reflecting a core BCT providing detailed user instructions on how to perform the target behavior, we created prompt user-guide pages when user registration is completed. This step-by-step, tutorial information clarifies the relational mechanism between the number of steps and the game time to solve puzzles as a reflective reward for the users.

### 5.2 Functional prototype of PuzzleWalk

**Fig 4:**
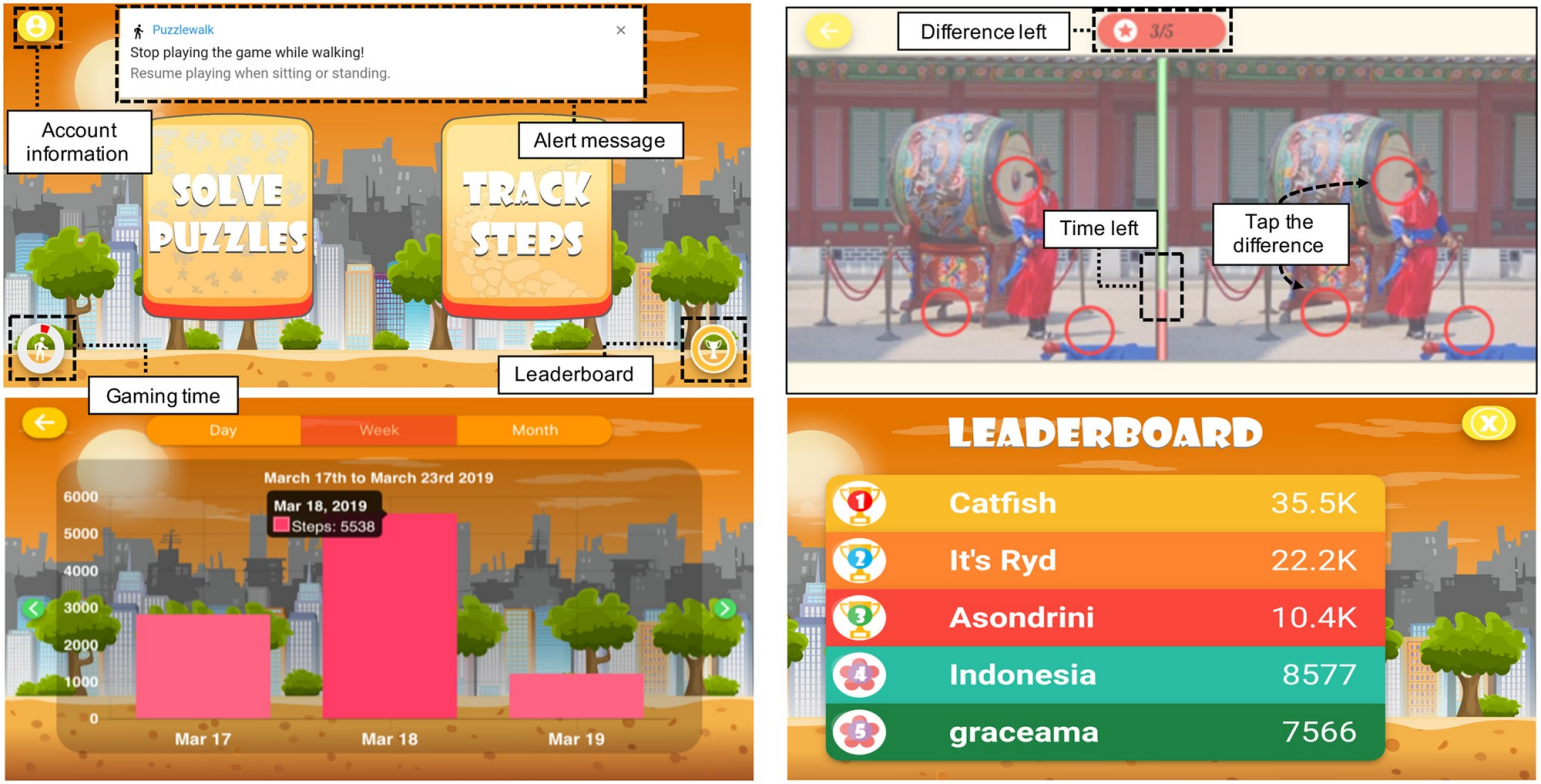
Screenshots of the functional prototype.

Based on the high-fidelity prototype and the iterative participatory design sessions from Phase 2, *PuzzleWalk* has gone through significant design changes that included revised gamification strategy, feedback mechanism, reward mechanism, and analysis of user and context requirements. Furthermore, we considered visual support, safety, and extrinsic motivation as pivotal factors that could result in sustainable impact on the PA of adults with ASD. Consequently, the functional prototype was developed with the “spot the difference” puzzles (Fig 4). Puzzle images consist of pictures of 100 cities all over the world to incentivize users to increase and sustain PA engagement. The design principle of the *PuzzleWalk* is the conversion between walking steps and puzzle-solving time. The accumulated walking steps will automatically be converted to puzzle-solving time so that users will be motivated to increase their walking steps to solve more puzzles. Overall, it took a total of 164 man-hours for a professional software development team to implement the functional prototype of *PuzzleWalk* (100 man-hours for implementing initial features, 28 for app refinement based on user feedback, 16 for troubleshooting and bug fixes, and 20 for app, server, and user account maintenance).

#### 5.2.1 How to play

Five minutes of puzzle-solving time are granted to the user every morning. A user’s walking steps are converted to puzzle-solving time (i.e., each step is equivalent to one second of puzzle-solving time). The more steps a user takes, the more puzzle-solving time will be given. Based on the calculated sum of puzzle points and accumulated walking steps, the leaderboard provides users with real-time rank information up to 15 users and the top three users receive a monetary reward every month.

## 6 Phase 3: Usability evaluation

Feedback that we received from the previous phases allowed us to create a functional prototype of *PuzzleWalk* that adults with ASD may want to use sustainably. The goal of Phase 3 was an evaluation of the usability. We recruited 18 participants (eight females; nine for non-ASD group, nine for ASD group) aged 20-40 (mean = 27). For the ASD group, our inclusion criteria were the same as Phase 1; nine non-ASD adults served as a control group to examine possible differences in their usability perceptions and perspectives on technology use, PA and sedentary behavior.

**Fig 5:**
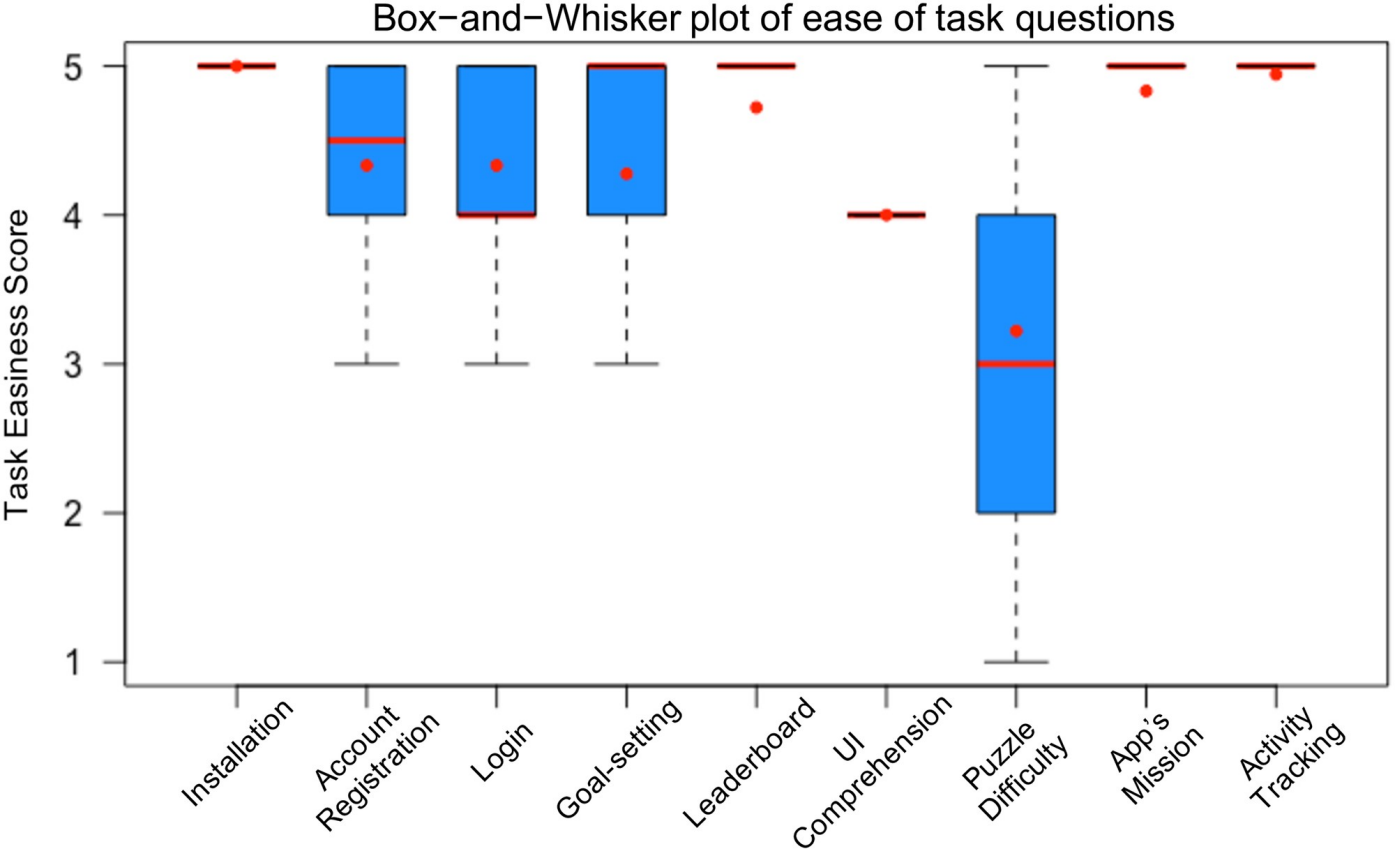
Cognitive walktrough results (1 = “very difficult” to 5 “very easy”; red lines = median and red dots = mean).

We conducted a short survey to examine participants’ demographics (i.e., gender, age, ethnicity, occupation, height, weight, frequency of smartphone use, medication). We then employed a think-aloud protocol [98] while they explored *PuzzleWalk*. We instructed the participants to follow each of nine primary functionalities of *PuzzleWalk* (i.e., search and installation, account registration, login, goal setting, leaderboard, UI comprehension, puzzle difficulty, app’s mission, and walking performance tracking) and describe their spontaneous perception of the app as they encountered each feature. Throughout the think-aloud session, the participants were also asked to answer one ease-of-task question (i.e., How easy or difficult did you find a certain task?) after they explored each functionality. This is based on the cognitive walkthrough method [99] to measure users’ perception of system usability on a 5-point Likert scale after the completion of each task. Afterwards, we asked the participants to complete a 10-item System Usability Scale (SUS) questionnaire [100, 101] and conducted the individual interviews to collect overall feedback of *PuzzleWalk* consecutively. The think-aloud sessions and individual interviews were audio-recorded and open coded by the three authors of this paper [102]. As shown in Fig 5, cognitive walkthrough results with ease-of task questions were analyzed by mapping with nine primary functionalities of *PuzzleWalk*.

### 6.1 Phase 3 findings: Cognitive walkthrough, think-aloud, and system usability scale

We asked each participant to complete a cognitive walkthrough, a System Usability Scale (SUS) questionnaire, and interviews in a consecutive manner. From this, we derived the participants’ evaluation on overall system satisfaction and usability, based on the quantitative and qualitative feedback collected.

For cognitive walkthrough results, the participants found the *PuzzleWalk* features very easy to use (see Fig 5). We conducted one-way ANOVA with Tukey’s HSD post-hoc analysis. We observed that significant variation existed across the nine ease-of-task responses (F(8, 153) = 10.8, *p* < 0.05). Results showed that puzzle difficulty is more difficult than other features. This result is consistent with our goal of making *PuzzleWalk* more challenging by creating three difficulty levels. The fact that our participants found *PuzzleWalk* to be of average difficulty shows that the difficulty level in *PuzzleWalk* is consistent with their cognitive abilities to solve the puzzle.

The think-aloud sessions confirmed that both ASD and non-ASD groups appreciate the design and practical use of *PuzzleWalk*. The participants showed great interest in visual functionalities (e.g., animated icons, leaderboard, and puzzle pictures of diverse countries). In particular, we found that the leaderboard provides a sense of belonging for the participants with ASD. From the individual interviews, we found that they are also very positive about the game feature and perceive *PuzzleWalk* to be less overwhelming compared to other games.

“The app is very cute. I like the colors, but it is not overwhelming. Usually there is a lot of specific nature scenery, so it feels like we are learning about another culture.”

(P3-3; an adult with ASD)

Moreover, we observed that adults with ASD wanted to fully understand *PuzzleWalk*. For example, they wanted to know exactly how much time was deducted and how the total score was calculated in the game. Although the non-ASD group mentioned that they passed the guide pages quickly without reading through them carefully, the adults with ASD group said they perused through guide pages in detail and found the guide pages very informative for understanding the game.

We examined whether the ASD group shows a statistically different SUS score than the non-ASD group. To do this, we conducted a normality test and a homogeneity of variance test due to the small sample size (n = 18). The Shapiro-Wilk normality test revealed that the values of both groups were normally distributed (*p* > 0.05). The Levene’s test revealed that the group variances are equal between ASD and non-ASD groups (*p* > 0.05). Based on these tests, we were able to apply paired t-test and showed that there is no significant difference (*t*(8) = 0.7, *p* > 0.05) between the means of the two groups (the ASD group: 85.83±14.51, the non-ASD group: 81.94±6.37) Thus, we concluded that both the general population and adults with ASD rated *PuzzleWalk* equally high in terms of usability.

Overall, the usability evaluation shows that adults with ASD found *PuzzleWalk* to be attractive, fun, and engaging. The satisfactory usability increases the likelihood that *PuzzleWalk* can be used as an effective PA intervention tool for them.

## 7 Phase 4: Perception of use and feasibility

In Phase 4, we focused on understanding the perception of use and feasibility of *PuzzleWalk* in a field study. We recruited 17 participants (11 males, 6 females) between the age of 18-42 (avg.: 28.75) again from popular online autism support groups, such as Wrong Planet, Reddit, and Facebook. Eligible participants were: (1) aged between 18-55 years old, (2) were diagnosed with ASD by a medical professional, (3) have access to Android or iOS smartphones, and (4) were physically and cognitively able to use the device (e.g., ambulate without an assistive device; understand purpose of the app). We collected their demographic information (i.e., gender, age, ethnicity, occupation, height, weight, medication, autism severity). We then conducted a screening interview (avg.: 12 minutes) to verify their eligibility. Nine participants were excluded because they could not complete the study requirements due to the severity of their ASD symptoms. In the end, eight participants were deemed eligible for the study.

The field study spans over a period of one month and included two data collection time points: (1) Week 1-3; baseline (without *PuzzleWalk*) and (2) Week 4 (with *PuzzleWalk*); intervention. In this study, we used a three-week baseline period under randomized control intervention. By doing so, we could have the opportunity to observe their PA patterns on a regular basis and screen participants’ compliance or responses before providing intervention. Also, we could verify functional stability of the developed PA-tracking app and process the randomization protocol. Eight participants were randomly assigned to one of two groups: a gamified, BCTs-based mobile app *PuzzleWalk* and a non-gamified, BCTs-based mobile app Google Fit. Instruction on how to use each app as well as a familiarization period for the app use was provided before the start of the intervention period. The participants additionally installed a mobile app developed by the research team that unobtrusively collects participants’ activity data (e.g., stationary and active/walking times) during the study period. We also asked an open-ended question at the end of the intervention period to gather overall feedback about *PuzzleWalk* and the field study.

### 7.1 Phase 4 findings: Field deployment

Given the relatively small sample size, we refrain from drawing statistical interpretations. We want to highlight that our goal for this field study is to understand the perception and feasibility of *PuzzleWalk* use to fine-tune the system and study designs for a larger-scale and longer-term study. The *PuzzleWalk* (PW) group showed an increasing trend in active/walking time while the Google Fit (GF) group remained similar to the baseline (see Table 2).

**Table 2:** Demographic information of the field study participants and active (walking) time. “B” = Baseline & “I” = Intervention.

	Demographic Information			Active Time (min/day)		
	Age	Sex	Education Level	B	I	% of Change
PW1	19	F	Some college	32.9	59.5	**81%**
PW2	42	F	4-yr college degree	22.8	27.4	**20%**
PW3	27	M	4-yr college degree	47.9	96.4	**101%**
PW4	31	M	High school/GED	13.6	14.4	6%
GF1	19	F	Some college	36.8	39.0	6%
GF2	35	M	4-yr college degree	42.1	20.7	-51%
GF3	31	M	Some college	103.0	98.4	-5%
GF4	18	M	High school/GED	60.7	52.7	-13%

From the open-ended question, we identified that the majority of participants found solving puzzles fun and showed a strong motivation to solve them. They said they wanted to save the game progress when they did not have enough puzzle-solving time or need to stop the game.

“I would like to be able to save my progress on the puzzles. It’s discouraging to be kicked off and unable to save my progress before I leave the screen.”

(P4-2; PW)

Despite a possible novelty effect during the relatively short intervention period, *PuzzleWalk* demonstrated the potential impact on increasing active/walking time in adults with ASD. Given the well-documented traits in this population-restricted and repetitive behavior and interest [1, 103], it is possible that adults with ASD could sustain behavior change for a longer period than the general population.

## 8 Design implications

### 8.1 Desire for social connection

Prior studies confirmed that people with ASD respond well to visual stimuli [40, 104, 105]. Participants in our study provided a lot of feedback on visual elements that were not typical for users without ASD. Among diverse design elements of *PuzzleWalk* for promoting PA (i.e., animated pictures for puzzles, the working step bar graph, leaderboard), the participants praised the leaderboard for providing a feeling that they belonged to a part of a community. This shows that although the ASD population typically struggles with communication, they do have a strong desire for socialization, and future designs for the ASD could emphasize the creation of visualization elements to foster the right balance of social connectedness vs. maintaining their personal autonomy.

### 8.2 Predictable system

We found that adults with ASD wanted to be fully aware of all the rules and processes in *PuzzleWalk*. The participants in the ASD group read the user guide pages about the game rules extremely thoroughly. The need for the well-articulated guide pages was identified when we conducted our participatory design sessions in Phase 2. This finding coincides with the fact that ASD population tends to have a strong preference for following rules and routines [1, 106, 107], and prefers mobile technologies with predictable and persistent interfaces [40]. Therefore, when designing the system for adults with ASD, clear instructions and orderly and predictable system interfaces are very important. For a game to provide successful PA intervention, game designers must design and propose clear game rules and processes to make PA part of their routines.

### 8.3 Focused and filtered information

Given the highly focused nature of the ASD population [108, 109], the majority of our participants stated that when an element is presented on the screen, they wanted to know information that is key and essential to the operating mechanism of *PuzzleWalk*. While playing *PuzzleWalk*, the participants were interested in information relevant to their gaming goal and performance (i.e., gaming time and high score). For example, in our high-fidelity prototype, we provided the walking distance, consumed calories, and walk time to present prompt self-monitoring of performance. However, the participants considered them as a distracting piece of information since it is unrelated to their overall objective (which is to obtain the highest possible score in the game).

### 8.4 Autism as a spectrum disorder

The participants in our study possess sufficient cognitive function to understand the process of our research and the *PuzzleWalk* app. While recruiting, we also met some applicants with relatively lower cognitive function for their age. Although our current system design did not address the population with severe autism, the design insights generated from this study could serve as an initial cue to understand how to encourage adults with ASD to participate in PA. Future research is necessary to extract user requirements and design insights from people with severe autism.

### 8.5 Maintaining progress

We observed that adults with ASD have a strong desire to save their progress for incomplete puzzles in *PuzzleWalk*. Prior research of the ASD population have demonstrated that adults with ASD show a pattern of overly regulated thinking, such as obsessions, intense interest, and a strong preference to maintain sameness [110]. In addition, incomplete puzzles can make them feel uncomfortable since they have a tendency to fixate on unfinished tasks repeatedly [111]. Therefore, the ability to save gameplay progress and resume it at a later time is an important game design element for people with ASD.

### 8.6 Limitations

The current study demonstrates several limitations. First, the small sample size of the field study makes it difficult to draw meaningful interpretation on *PuzzleWalk*’s impact. As such, this paper focuses on reporting the design rationales and lessons learned throughout the design inquiry, and we leave it to future studies to assess the long-term sustainability of a mobile app-guided PA intervention in adults with ASD. Second, participants in the survey study were comprised of adults with ASD recruited from online autism advocacy/support groups. The study findings may not be generalizable to those who are less familiar or comfortable with technology use. Another underlying limitation lies in the retrospective-reporting method for PA assessment. We used the IPAQ short form questionnaires in Phase 1 to ask the participants’ last week PA, and the self-report of PA outcomes could suffer from recall bias. Given the difficulty of reporting one’s PA status in a retrospective manner, ecological momentary assessment (EMA) method is a promising method to mitigate this challenge. We plan to apply EMA in future studies [112, 113].

## 9 Conclusion

We propose the app as a readily applicable and adjunct intervention option for increasing PA, and, consequently, reducing anxiety in adults with ASD. The four overarching system development phases provide practical insights into the ongoing challenges and facilitators of the mobile app-guided PA intervention, as well as critical considerations for adaptable and sociotechnical game and intervention design for adults with ASD focusing on visual communication, gamified motivation strategies, and BCTs. This work focuses on illuminating the design rationales and lessons learned for designing a mobile health game for adults with ASD. We are in the process of conducting a larger scale field study over a longer period to assess the impact of *PuzzleWalk* on PA and applying EMA to collect more accuracy reporting of anxiety outcomes.
